# A Review of Dissimilar Welding Techniques for Magnesium Alloys to Aluminum Alloys

**DOI:** 10.3390/ma7053735

**Published:** 2014-05-08

**Authors:** Liming Liu, Daxin Ren, Fei Liu

**Affiliations:** Key Laboratory of Liaoning Advanced Welding and Joining Technology, School of Materials Science and Engineering, Dalian University of Technology, Dalian 116024, Liaoning, China; E-Mails: rendx@dlut.edu.cn (D.R.); liufei33733@163.com (F.L.)

**Keywords:** review, aluminum alloys, magnesium alloys, welding, intermetallic compound, mechanical property

## Abstract

Welding of dissimilar magnesium alloys and aluminum alloys is an important issue because of their increasing applications in industries. In this document, the research and progress of a variety of welding techniques for joining dissimilar Mg alloys and Al alloys are reviewed from different perspectives. Welding of dissimilar Mg and Al is challenging due to the formation of brittle intermetallic compound (IMC) such as Mg_17_Al_12_ and Mg_2_Al_3_. In order to increase the joint strength, three main research approaches were used to eliminate or reduce the Mg-Al intermetallic reaction layer. First, solid state welding techniques which have a low welding temperature were used to reduce the IMCs. Second, IMC variety and distribution were controlled to avoid the degradation of the joining strength in fusion welding. Third, techniques which have relatively controllable reaction time and energy were used to eliminate the IMCs. Some important processing parameters and their effects on weld quality are discussed, and the microstructure and metallurgical reaction are described. Mechanical properties of welds such as hardness, tensile, shear and fatigue strength are discussed. The aim of the report is to review the recent progress in the welding of dissimilar Mg and Al to provide a basis for follow-up research.

## 1. Introduction

### 1.1. Background

Aluminum and its alloys are widely used in the automotive industry because of their low density, high specific strength, good corrosion resistance, good workability, high thermal and electrical conductivity, attractive appearance, and intrinsic recyclability [1–4]. As an extremely light metal, magnesium and its alloys have excellent specific strength, excellent sound damping capabilities, good castability, hot formability, good electromagnetic interference shielding, and recyclability [5–12]. Table 1 compares the typical physical properties of pure magnesium, aluminum and iron [13]. It can be seen that using Al alloys and Mg alloys can provide great weight savings compared to steel and cast iron.

There are certain applications for which one of the metals is more suitable than another due to advantages of a specific property, such as damping capacity for Mg and creep resistance for Al. The both Mg and Al are expected to be used in industry in the future. Combining Al and Mg in one hybrid structure would make possible the use of these alloys for even more applications which will result in desirable weight saving. With the increased use of Al and Mg, there is a pressing need for a technology to produce dissimilar Al/Mg joints. Due to continuously increasing usage of Al and Mg in industry and the large amount of potential applications of Al/Mg hybrid structures, the problem of joining these materials has to be solved.

### 1.2. Research Status

**Figure 1.**
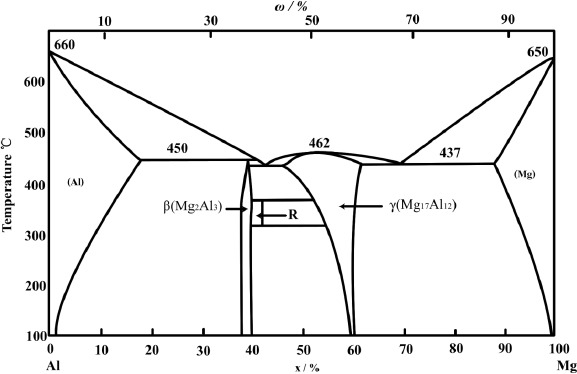
Al-Mg binary phase diagram [15].

The major difficulty in the welding of Al and Mg is the formation of hard and brittle intermetallic compounds (IMCs) which have a detrimental effect on the joint’s strength [14]. Numerous investigations regarding joining of dissimilar Al and Mg by different techniques can be found in previous studies. According to the Al-Mg binary phase diagram (see Figure 1) [15], intermetallic compounds such as Mg_2_Al_3_ and Mg_17_Al_12_ can be formed in the weld after solidifying [16]. The microhardness of IMC ranges from HV 152 to HV 221 depending on the location in the interface, while the base metals of Al and Mg have average hardness values from HV 25–60 [17]. Noticeable higher hardness of the interface than of the Al and Mg base metals confirms that the high hardness Al-Mg IMCs have been formed within the Al/Mg interface, which results in low strength in fusion welding. The tungsten inert gas arc welding (TIG) directly welded Mg/Al joint presented a very undesirable strength, and the maximum shear strength of the laser welded joint could only reach 48 MPa [18].

The technological difficulty in the joining of Mg/Al being dissimilar metals is mainly caused by the brittle intermetallic reaction layer formation in the weld. There, the key to increasing the joint strength is to control the IMCs to eliminate or reduce the negative effect, and until now, three main research approaches have been used. First, techniques with low welding temperature were used to reduce the IMCs. By this approach, solid state processes which involve comparatively low temperatures, such as friction stir welding and diffusion bonding, can achieve relatively high strength. Second, IMC variety and distribution were controlled to avoid the degradation of joining strength in fusion welding. By this approach, employing filler metals to further alloy the weld seam was used in laser welding, arc welding, and laser-arc hybrid welding. Meanwhile, weld bonding, including laser weld bonding and friction stir spot adhesive bonding, were used to join Mg and Al. The adhesive can improve the microstructure and mechanical performance of Al/Mg welds by the stirring effect on the molten pool. Third, explosive welding, ultrasonic welding, resistance spot welding and electromagnetic impact welding were used to reduce the IMCs because these techniques have relative short reaction time and low energy. The Mg/Al joint strength has been significant improved, and some of the techniques are very promising for joining Mg and Al alloy in the work place. The detailed research progress and results of these techniques are reviewed in the following sections using different approaches.

## 2. Solid State Processes

### 2.1. Friction Stir Welding

Friction stir welding (FSW) is a solid-state welding process in which the pin at the bottom of the rotating tool is plunged into the workpiece and traversed along the joint to cause bonding by stirring and mixing [19]. FSW can be used to join dissimilar as well as similar metals. Some early papers [20–22] examined microstructural features in dissimilar FSW of Al alloy 1050 and Mg alloy AZ31, and discussed microstructural evolution. In the later study, some more dissimilar Mg alloys and Al alloys were weld by FWS, and microstructures, strength, effect of parameters and the intermetallic formation mechanism were investigated [23,24].

#### 2.1.1. Microstructures

**Figure 2.**
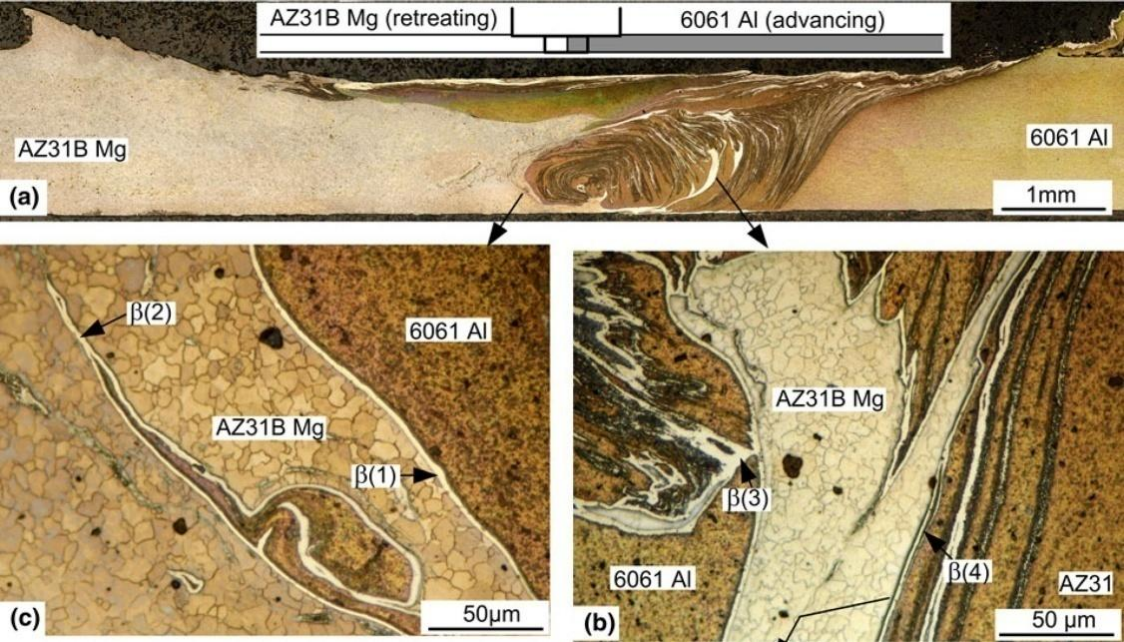
Macrostructures of friction stir welded Mg-Al joint [19]. (**a**) A transverse macrograph; (**b**) and (**c**) transverse micrographs showing Al_3_Mg_2_

As shown in a typical transverse macrograph of butt FSWed joint (see Figure 2), a complex flow pattern characterized by intercalation lamellae is formed in the stir zone [19,23,24]. Dynamic recrystallization (DRX) was observed in the weld region as well as in the transition region, with a clear decrease in the grain size from the base materials through the transition zone and into the weld zone. This recrystallization was enabled by frictional heat from the tool shoulder and tool probe, heat generated by the mechanical stirring of the materials by the probe and mostly adiabatic heat contributing to DRX through the deformation.

**Figure 3.**
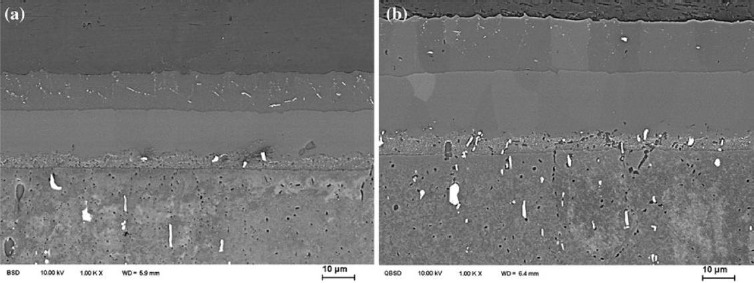
Microstructures of intermetallic compounds (IMCs) in friction stir welded Mg-Al joint [28]. (**a**) Optical micrograph; (b–g) images of intermetallic compounds.

Friction stir welding has a lower input compared with fusion welding. However, it was found that the formation of Mg_2_Al_3_ and Mg_17_Al_12_ IMCs are inevitable in the dissimilar Al/Mg joints under all conditions of the welding. In solid state, wetting of Al/Al grain boundaries (GBs) by the second solid phase Al_3_Mg_2_ has been observed as reported [25,26]. Below the tie line at T_wsmin_ = 220 °C, no Al/Al GBs wetted by the second solid phase Al_3_Mg_2_ were present in the polycrystals. Above the tie-line at T_wsmax_ = 410 °C all Al/Al GBs were wetted by the second solid phase and separated from each other by the continuous Al_3_Mg_2_ layers. In the FSWed Al/Mg joint, a characteristic interfacial layer consisting of IMCs Mg_2_Al_3_ and Mg_17_Al_12_ was commonly observed between the Al and Mg alloy [20,24,27–29] as shown in Figure 3. The layer on the Al side has been identified as Mg_2_Al_3_ and that on the Mg side as a eutectic consisting of Mg_17_Al_12_ and the Mg-rich phase. TEM of the stir zone show that the Mg_2_Al_3_ layer consists not only of fine Mg_2_Al_3_ grains but also nanosized Al particles embedded in them [28], and these fine grains and nanosized particles are likely to be produced by the shearing action during solidification. The presence of these microstructures in the stir zones of lap and butt welds is clear evidence of liquid formation during FSW.

#### 2.1.2. Formation Mechanism of IMCs

FSW was used to eliminate the intermetallic reaction layer, but the IMCs could only be reduced. Formation mechanisms of liquid and intermetallics were investigated by some researchers, and the thermal behavior during the FWS was measured. The measured temperature, resulting in the vicinity of the tool, showed that the peak temperature corresponded to reference [30] and higher [31] (450 °C) than the equilibrium solidus temperature of the eutectic structure (437 °C). Firouzdor found that the peak temperature was slightly below the eutectic reaction because the thermocouples were pushed downward during welding [28]. They also confirmed that the solidified droplets melted at 436 and 449 °C by differential scanning calorimetry, nearly identical to the eutectic temperatures.

At the beginning of the FSW process, due to the frictional heating, the weld is heated to a peak temperature of 450 °C, which is higher than the equilibrium solidus temperature of the eutectic structure. The plastic deformation and high-temperature exposure induced the grain boundary diffusion and the interfacial diffusion, thus local melting occurred. Liquefaction and solidification occurred repeatedly, resulting in a non-equilibrium solidus temperature. During the sleeve retraction period, the “solid-liquid” phase material experienced further diffusion and dynamic recrystallization, resulting in the formation of the fine equiaxed Mg_17_Al_12_ grains at the weld center [31].

**Figure 4.**
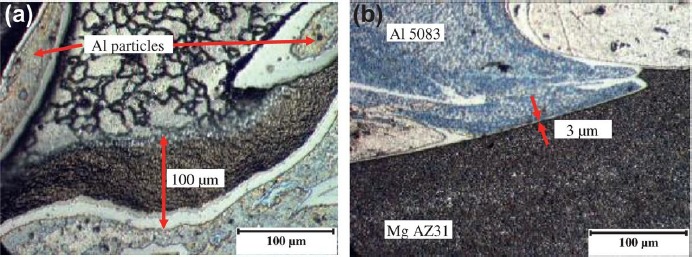
Microstructure of Al/Mg interface for (**a**) friction stir welded specimen in air and (**b**) submerged friction stir welded specimen under water.

**Figure 5.**
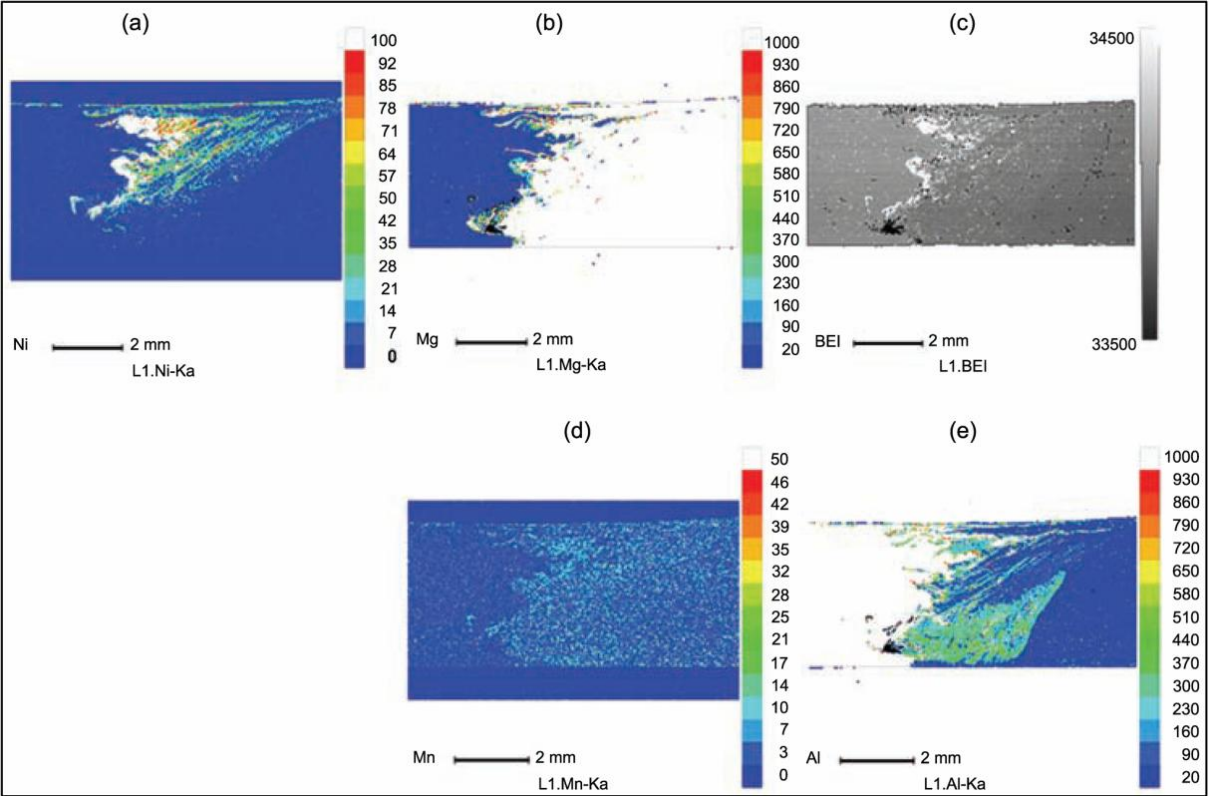
EPMA (Electron probe micro-analyzer) image and distribution maps of major elements of the dissimilar hybrid welded Al/Mg alloy with Ni-foil as interlayer [33]. (**a**) The distribution map of Ni; (**b**) the distribution map of Mg; (**c**) EPMA image; (**d**) the distribution map of Mn; (**e**) the distribution map of Al

Since the IMC cannot be eliminated, some advanced FSW processes have been used to further reduce the harmful influence of the IMC. Al alloy to Mg alloy were friction stir welded with water cooling to suppress the formation of IMCs by increasing the cooling speed [32]. It was found that due to a decrease of 25 °C in the peak temperature from a maximum of 403 °C in the case of an air welded specimen to a maximum of 378 °C for welds made underwater, much lower amounts of IMCs were formed during FSW under water as shown in Figure 4. Laser hybrid friction stir welding was used to join Al alloy to Mg alloy with Ni foil as filler material [33]. The strength may be increased due to the presence of less brittle Ni-based intermetallic phases instead of Al_12_Mg_17_. Ni was distributed well at the interface of the Mg and Al alloy as shown in Figure 5, and the distribution considerably reduced the formation of brittle Al-Mg IMCs.

#### 2.1.3. Mechanical Properties

Heavy thickness of the IMC layer seriously deteriorates the mechanical properties of the joints [19,20,23,24,27,29,34]. In order to improve the strength of the FSWed joints, the effects of parameters, such as material position, travel speed and rotation speed were investigated. The heat input is a key variable governing the effect of the welding conditions on the joint strength. The heat input significantly affects the formation of IMCs and material flow. The thickness of the IMC layer increases with increasing tool rotation speed and decreasing welding speed, which are related to higher input [19,28,33,35]. The FSWed Mg/Al joint strength can be improved by reducing the heat input and hence the detrimental formation of intermetallic compounds and liquid films. However, low input does not always result in high joint strength, and higher strength in the FSWed Al/Mg joint is promoted by mechanical interlocking through production of complex weld interfaces [19]: strength increases with increasing interface length, interpenetrating feature thickness. The Al/Mg dissimilar butt FSWed joint exhibited an s-shaped interface at lower rotational speeds, and at higher rotational speeds, the weld joints develop an interpenetrating feature along with the curved interfaces. The tensile failure of the weld joints occurs by two mechanisms: fracture along the IMCs, where the continuous layer is observed, and through the aluminum base metal in the interpenetrating feature regions [36]. In lap welding, defect-free welds were successfully obtained and the surface morphology of the welds became smoother as the tool rotation speed was increased [35]. The shear strength was higher when the region composed of IMCs was mixed with a region containing the α-Mg+Mg_17_Al_12_ eutectic structure, resulting from higher heat input parameters [29]. Chen also reported that using a lower welding speed results in no visible welding cracks in the joint and improves the joint strength [37].

Through parameter optimizing, the strength of the direct FSWed joint was improved. Al-to-Mg butt welds can be made by butt FSW with good joint strength up to 80% to 100% of the joint strength of Mg-to-Mg welds [19] and about 132 MPa, which was about 66% of the tensile strength of the A5052P-O alloy [35]. Tensile strength of the joint reached about 168 MPa tensile strength in the case of hybrid welding with Ni foil and showed a higher value than that of the friction stir welded joint with and without the Ni foil [33]. The lap shear strength and fatigue life of the friction stir spot welded joint with and without adhesive were also tested; the result showed that the added adhesive can significant improve the tensile shear strength and failure strength of the Al/Mg dissimilar weld whose values were significantly lower than those of the similar joints of Mg/Mg and Mg/Al [38,39].

### 2.2. Diffusion Bonding

**Figure 6.**
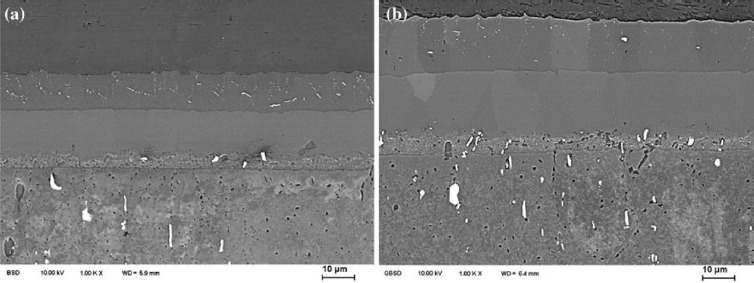
Microstructures of Al/Mg diffusion welded joint at different temperatures [42]. (**a**) Diffusion-welding at 703 °C for 1200 s; (**b**) diffusion-welding at 703 °C for 1800 s

Diffusion bonding is a typical solid state technique which is suitable for joining dissimilar materials. The predominant process parameters in diffusion bonding process are: temperature, pressure, time and surface roughness. It has been found that bonding temperature has a greater influence on shear strength and bonding strength of the joints followed by bonding pressure, holding time, and surface roughness [40]. Different microstructures formed under different parameters. However, the results showed that the presence of intermetallic compounds was confirmed even when the parameters were optimized [41,42]. The diffusion zone is composed of a Mg_2_Al_3_ layer and a Mg_17_Al_12_ layer as shown in Figure 6 [42]. As anticipated, the ductility of the intermetallic phases is remarkably lower than the base materials. A maximum shear strength of 57.70 MPa could be attained under the optimized bonding conditions as reported [40].

**Figure 7.**
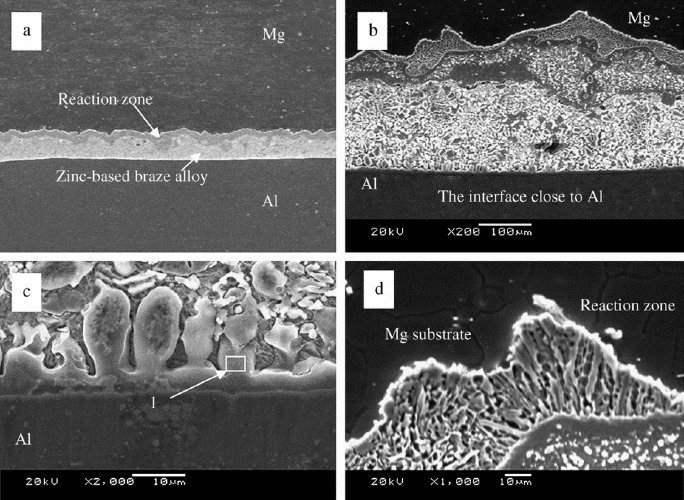
Microstructure of the brazed joint with Zn-1.5Al-2Re interlayer [43]. (**a**) Cross section of the joint; (**b**–**d**) details of the reaction zone.

As mentioned above, formation of IMCs was inevitable in direct fusion bonding because the Mg-Al reaction temperature is relatively low. There, some filler metals were set as a barrier interlayer between the dissimilar metals avoiding the formation of Mg-Al IMCs. Zn and Zn-based alloys were mainly selected as the filler metal for welding of Mg and Al because Zn has a relatively low melting point. Furthermore, Zn can form a solid solution with Al and does not form intermetallic compounds, which may somewhat enhance the property of the joints. Zn has the same crystal lattice with Mg, and they together can form a variety of intermetallic compounds, such as MgZn, Mg_2_Zn_3_, MgZn_2_ and Mg_2_Zn_11_. Al alloy was bonded to Mg alloy successfully using zinc-based alloys as interlayer [43–46], and the effect of interlayer compositions, thickness, bonding temperatures and times on the microstructure characterization and strength were investigated. The experimental results showed that Mg-Al IMCs were avoided for the addition of the zinc-based alloy, and the Mg/Al joint strength could be significantly improved. Liu *et al.* [43,44] found that when Zn-1.5Al-2Re was used as interlayer, the Mg substrate and the remanent filler alloy were bonded with the reaction zone that formed along the zinc rich and magnesium poor interface as shown in Figure 7. Only a few Mg-Zn intermetallic compounds (MgZn_2_) existed homogeneously in the reaction zone. The substrate and the remanent brazing alloy were bonded with a thin Al-Zn solution. Zhao *et al.* [45,46] investigated diffusion bonding of Mg and Al alloys with different interlayer compositions, where the interlayer was prepared by a hot dipping technique in pure Zn, Zn-8Al and Zn-5Al baths, respectively. It was found that the interlayer of Zn-5Al led to the formation of an interface microstructure composed of mini Al-rich particles dispersed in a MgZn_2_ phase. The dispersive soft Al-rich phases especially, impeded the expansion of fracture in the base MgZn_2_ phase, and improved the joint strength from 41 to 83 MPa.

Sn also has a relatively low melting point and can interact with both Mg and Al, so Sn and Sn-Zn interlayers have been used to weld Mg and Al [47,48]. The addition of Sn and Sn-Zn interlayers were both observed to successfully eliminate the brittle Mg_17_Al_12_ and Mg_2_Al_3_ IMCs, which were replaced by the new formed composite layer, resulting in a significant improvement in joint strength. However, Sn and Mg_2_Sn were located on both the Al and Mg sides of the matching fracture surfaces indicating that the tensile shear failure occurred through the interior of the interlayer [47]. The formation of Mg-Sn IMCs in the center of the bonding zone becomes the key to the strength. There, addition of Ce element to Sn-30Zn solder is expected to depress the negative effects of blocky Mg-Sn IMCs and further optimize the Mg/Al joint. Adding the appropriate amount of Ce was conducive to decreasing the amount of Mg_2_Sn IMCs and increasing the amount of Al-Sn-Zn solid solutions; the added Ce can increase the strength to 77.48 MPa [48].

Some other studies also indicate that Al alloy already was successfully joined to Mg alloy with a silver and Ni interlayer. The interfacial microstructure and mechanical performance of Mg/Al joints were greatly improved in the presence of the silver interlayer, which prevented the formation of brittle Mg-Al compounds [49]. The Mg-Al intermetallic compounds were eliminated owing to the presence of Mg-Ag compounds, and the typical microstructure at the joint interface was a Mg/Mg_3_Ag/MgAg/Al multilayer structure. Diffusion bonding of Al and Mg using a Ni interlayer was investigated, and the results showed that the Mg-Al intermetallic compounds were impeded [50]. The fracture occurred in the IMC layer of the Mg-Ni reaction layer, where the Mg_2_Ni phase appeared on both the Al and Mg surfaces.

Diffusion bonding can control the weld microstructures relatively precisely by setting the temperature, pressure and time, as well as controlling the thickness of the reaction layer. There, it is a reasonable means for improving the properties of Mg/Al joints by avoiding the formation of brittle Mg-Al IMCs. The reaction of Mg-Al can be hindered by the added interlayer, so Mg-Al IMCs can be avoided. The microstructure of the diffusion bonded joints was improved and the strength was increased. However, some other undesired brittle intermetallic compounds formed during the welding process because of the active metallurgical properties of Mg and Al, and these newly formed IMCs restricted the strength. In the future, the composition of the added interlayer should be optimized. Improving the microstructures of newly formed IMCs, such as Mg-Ni, Mg-Zn and Mg-Sn, can further increase the joint strength.

## 3. Fusion Welding

Some significant research studies, such as laser welding by FEM analysis [51], cold metal transfer MIG welding [52,53], linear friction welding [54] and diffusion welding [15], were carried out to improve the strength of the Mg/Al joint. However, it was noted that direct fusion welding, or even solid state welding, of Mg and Al offered lower strengths than expected because of the inevitable continuous IMC layers. The IMC variety and distribution were controlled to increase the mechanical performance of Al/Mg fusion welds.

### 3.1. Welding with Filler Metals

**Figure 8.**
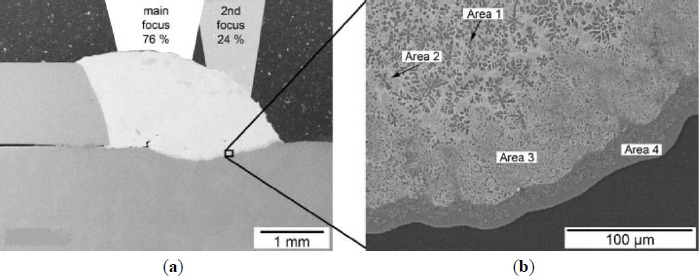
Cross-section and microstructures of laser welded joint with ZnAl15 [56]. (**a**) Overview; (**b**) detail of intermetallic phase seam

Zn based filler metals were also used in fusion welding such as arc welding and laser welding. Zhang *et al.* [55] investigated the microstructural evolution of Al/Mg lap joints welded using the MIG process with Zn foil as an interlayer. They found that the fusion zone has an Al-Si hypoeutectic structure and an Al-Zn eutectic structure. Multiple Al-Zn intermetallic compounds are found at the interface between the fusion zone and the unmelted magnesium base metal. The melted zinc foil formed a barrier that prevented the mixture of the fusion zone and the Mg base metal at the weld toe. Laser welding of Mg alloy and Al alloy was investigated by Scherm *et al.* [56], using a Nd:YAG laser with two focus optics and a ZnAl filler material as shown in Figure 8. Three different ZnAl alloys with 4, 10 and 15 wt% Al were investigated, and they found that the strength of the joint was significantly affected by the Al content of the filler wire: the higher the Al concentration, the higher the load to failure. The microstructure and strength results showed that a thin intermetallic phase seam did not accord with a higher strength of the joint, and the strength depends more on the depth of the weld penetration.

**Figure 9.**
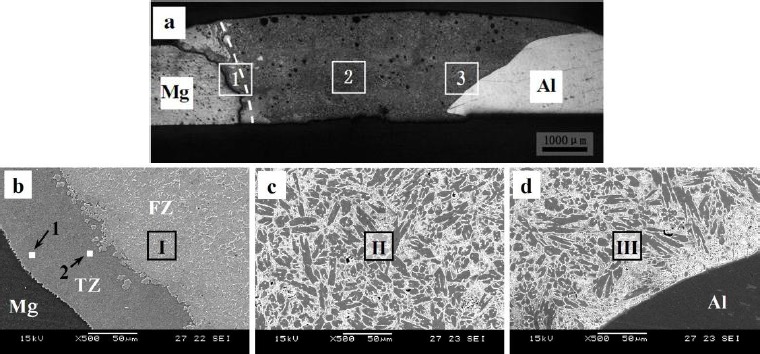
Microstructures of TIG welded Mg/Al joint filling with Zn filler metal [57]. (**a**) macrostructure of the joint; (**b**–**d**) the magnification of 1, 2 and 3 in (**a**).

**Figure 10.**
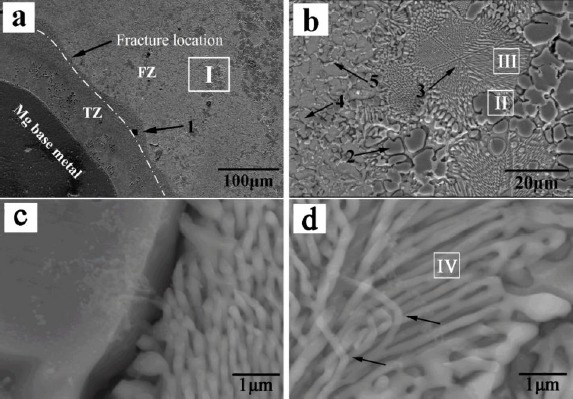
Microstructures of the weak zone of the TIG welded joint with Zn-29.5Al-0.5Ti filler metal [61]. (**a**) The macrostructure near Mg base metal; (**b**) the magnification of area I from (**a**); (**c**) and (**d**) the magnified images of area II and III in (**b**).

Liu *et al.* [57] investigated TIG welding of Mg and Al with Zn based filler metal. The results showed that when pure Zn was added, the mixture of Zn-based solid solution and Al-based solid solution (MZAS) with a continuous layer of MgZn2 IMCs in near the Mg base metal (see Figure 9) was the weakest area of the joint. To improve the tensile strength of the joint, a series of Zn-xAl filler metals was designed to accurately modulate the microstructure and composition of the alloyed welding seam, aiming to increase the amount of Al-based solid solution in the weak area of the FZ. The maximum average strength achieved was 120.1 MPa using Zn-30Al filler metal [58,59]. Fine grain strengthening is a common method to increase the property of materials, and rare-earth and Ti are great elements for Mg alloys and Al alloys [43,46,47,60]. There, Liu *et al.* [61,62] investigated the effects of Ce and Ti on the microstructures and strength, and the results showed that Ti can effectively increase the Mg/Al joint strength to 148 MPa with a Zn-29.5Al-0.5Ti filler metal. The addition of Ti can improve the microstructure of the FZ as shown in Figure 10, for the reason that the Al_3_Ti precipitated first due to the Gibbs energy of formation (ΔG) of Al_3_Ti which is much lower than that of Al_3_Mg_2_, MgZn_2_, and AlTi, and acted as the nucleus of heterogeneous nucleation during the solidification of the welding pool.

**Figure 11.**
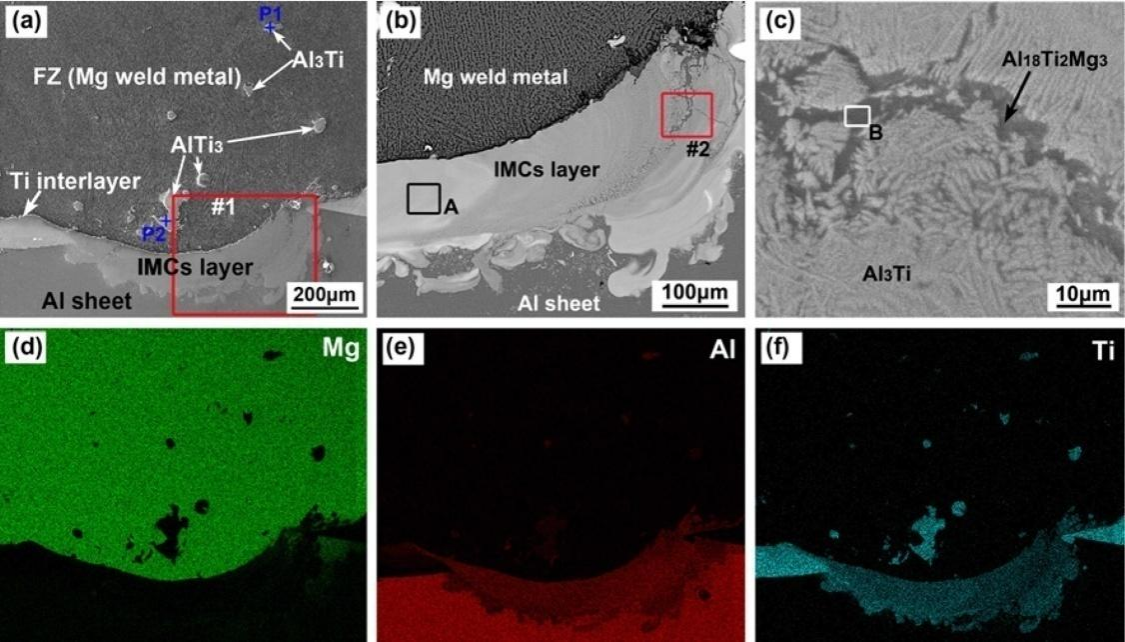
Element distribution maps of the laser welded Al/Mg joint with Ti-foil as interlayer [68]. (**a**) Overview of interfacial layer and the image for distribution maps of major elements; (**b**) details of area #1; (**c**) details of area #2; (**d**) map of element Mg; (**e**) map of element Al; (**f**) map of element Ti.

Untill now, it has been difficult to find one metal which can both react with Mg and Al, but not form intermetallics with either of them. Ti and Fe have similar properties in welding Mg and Al as interlayers. Ti and Fe can both react with Al to form IMCs, such as Ti_3_Al, TiAl, TiAl_3_, Fe_3_Al, FeAl and FeAl_3_. The considerable melting point difference and almost no intersolubility between Mg/Fe or Mg/Ti can be attributed to the marked differences in the metallurgical and physical properties between them [63–66]. In addition, similar to the idea of Penner *et al.* [67], metals with high melting points can entirely prevent the formation of Al-Mg intermetallics. Ti was used as an interlayer to join Mg and Al alloys by fiber laser welding [68]. The formation of Mg-Al IMCs could be totally suppressed and the interfacial layer was composed of Al_3_Ti with small amounts of Al_18_Ti_2_Mg_3_ as shown in Figure 11. The suppression of the Mg-Al IMCs helps to increase the tensile strength of the Mg-Al joints because Al_3_Ti is stronger than Mg-Al IMCs. The mechanism of interfacial layer formation was attributed to the thermodynamic behavior of the formation of IMCs in the Al-Mg-Ti ternary system and to precise control of the laser power. Mg alloy and Al alloy were lap joined together with the addition of Fe interlayer by hybrid laser-TIG welding [69]. The addition of Fe interlayer suppressed massive production of Mg-Al IMCs but produced Fe-Al intermetallics in the fusion zone of the joints. Deeper penetration inside the Al alloy contributes to the improvement of the shear strength of Fe-added joints, and the maximum shear strength of Fe-added joints could reach 100 MPa.

IMC variety and distribution were controlled to increase the mechanical performance of Al/Mg fusion welds. By this approach, filler metals were used to further alloy the weld seam to improve the microstructure in laser welding, arc welding and laser-TIG hybrid welding. Until now, the used filler metals include pure Zn, Ti, Fe, Sn, Ni, and some binary alloys or ternary alloys, such as Zn-Al alloys, Sn-Zn alloy, Al-Si alloy, Zn-Al-Ti alloys and Zn-Al-Ce alloys. These filler metals can significantly improve the microstructures of the Mg/Al joint, and reduce the degradation of the joining strength in fusion welding. However, it is difficult to find one alloy which can completely eliminate IMCs by reacting with Mg and Al in the weld. The newly formed IMCs such as Mg-Zn, Al-Fe and Al-Ti become the key factor in improving joint strength, and in the future some new alloys should be researched to solve this problem. Some more ternary alloys may be used to improve the microstructures by second phase strengthening or fine grain strengthening. Additionally, high-entropy alloys (multicomponent alloys) may be one proper choice for welding Mg and Al to avoid IMCs. High-entropy alloys containing a higher number (five or more than five) principal elements will more easily yield the formation of random solid solutions with simple bcc and fcc structures, rather than intermetallic compounds.

### 3.2. Weld Bonding

**Figure 12.**
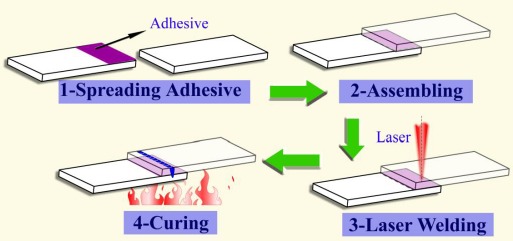
Laser weld bonding process [74].

The thickness and the distribution of the intermetallics have obviously an influence on the property of the Mg/Al joint, which is influenced by the behavior of the fusion zone. In order to regulate the distribution of the intermetallics, the laser weld bonding technology was used to join Mg to Al alloys. Weld bonding is an advanced hybrid joining technology which combines welding with adhesive bonding. Weld bonding can offer many benefits of both welding and adhesive bonding [70,71], and laser weld bonding has also been used for joining Al alloys and Mg alloys [72–74]. Based on the advantages of weld bonding, laser weld bonding is a promising process for joining Mg alloy and Al alloy. This process consists of four stages as follows: (1) spreading adhesive on the lower sheet surface; (2) applying pressure and assembling; (3) laser (spot) seam welding and (4) curing, as shown in Figure 12.

Mg alloy and Al alloy were joined successfully by laser seam weld bonding [75–78], and the differences between laser welding (LW) and laser weld bonding (LWB) were compared and discussed. It was found that LWB specimens gave higher tensile shear resistance because of the hybrid effect of laser welding and adhesive bonding. The adhesive would be decomposed during the welding process, which makes the intermetallics relatively dispersed. With the effect of the thermal stress and the characteristic of the Mg-Al intermetallic phase and the Mg-Mg_17_Al_12_ eutectic phase, the feasibility of the formation of the microcracks in the LWB Mg-Al joint would be obviously less than that in the laser welding joint [79]. At the same time, the gasification of the adhesive caused a baffle effect on the diffusion between the Mg and Al alloy. There, the thickness of the intermetallic compounds was reduced, which improved the LWBed joint [80,81].

Except for LWB technology, the friction stir spot adhesive welded method is used to join Mg to Al alloys. Chowdhurya *et al.* [39] investigated the lap shear strength and fatigue behavior of the friction stir spot adhesive welded AZ31B alloy to 5754 alloy and compared the results with those for the FSSWed joints. The result showed that the extent of forming IMCs decreased in the dissimilar adhesive joints in comparison to the Al/Mg weld without adhesive. The adhesive during the welding process suppressed the formation of intermetallic compounds.

It has been reported that the tensile shear load of the LWB Mg to Al joint was about 2.8 KN/cm after the adhesive cured, which is obviously higher than the laser welded joint [18,51]. However the increase of the property is mainly based on the adhesive bonding, and the welding joint tensile shear load without adhesive curing is about 1.0 KN/cm. A few Mg-Al intermetallics are still found in the fusion zone, producing some welding defects and decreasing the simple, laser weld bonding of the Mg to Al joint. There, the improvement of the simplified laser weld bonding joint is the main problem for LWB Mg to Al technology.

**Figure 13.**
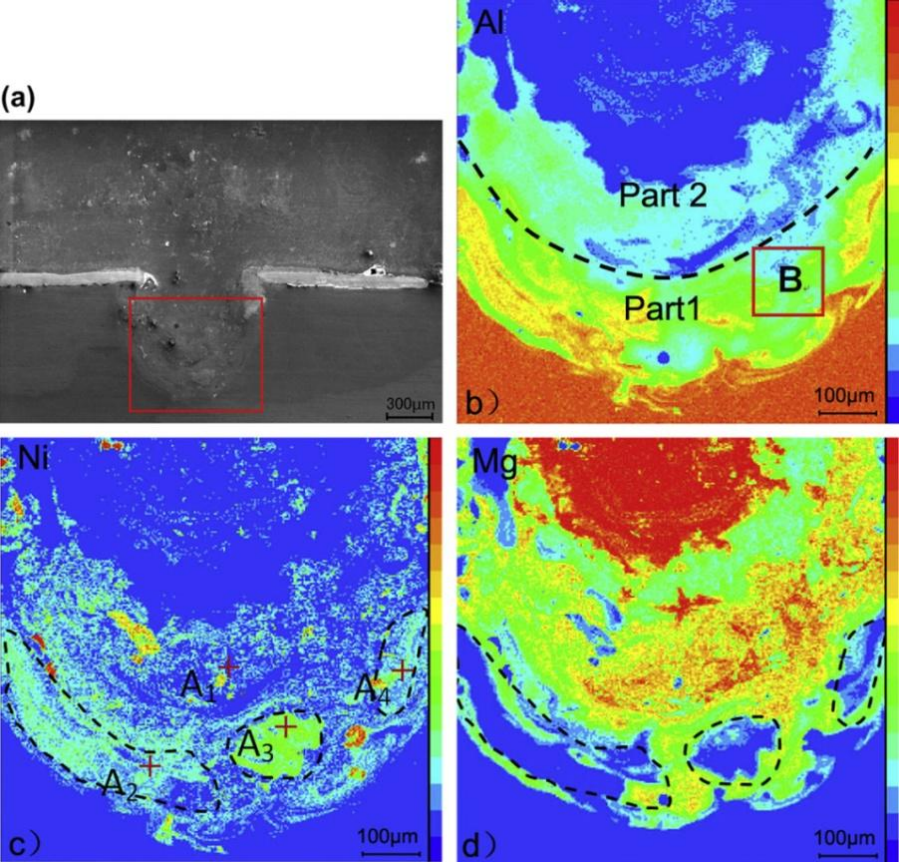
The macrostructure and element distributions of the laser-arc-adhesive hybrid welding of Mg to Al joint with Ni interlayer [83]. (**a**) Macrostructure; (**b**) element Al; (**b**) element Ni, (**d**) element Mg.

In order to improve the property of the simplified laser weld bonding joint, AZ31B Mg alloy and 6061Al alloys are joined by the laser weld-bonding technique with a galvanized iron interlayer [82]. Diffusion between Mg and Al is almost prevented by the iron interlayer, which is avoided in the formation of Mg-Al intermetallics. Only traces of Al-Fe IMCs were found at the bottom of the fusion zone. Similar alloying methods in weld bonding were also tried using Ni as interlayer [83]. The results showed that the adhesive and Ni interlayer restrain the reaction between Mg and Al as shown in the element distributions map of Figure 13. The transition zone between Mg and Al is composed of the Mg-Mg_2_Ni eutectic and Al-Al_3_Ni peritectic according to the analysis of the thermodynamic behavior. The function of the Ni interlayer on the fusion zone was strengthened with the addition of the adhesive. The tensile shear test load of the laser-arc-adhesive hybrid welding of Mg to Al joints is 1.7 KN/cm and 118 MPa without curing the adhesive. The harmful effects of the IMCs were significantly reduced and the property of the joint was improved.

In LWB and friction stir spot adhesive bonding process, the decomposition of the adhesive changes the flow behavior of the fusion zone and the diffusion between the Mg and Al elements, which influence the distribution and the thickness of the Mg-Al intermetallics and improve the property of the joint. On the other side, as the adhesive does not react with the Mg or Al alloys, the Mg-Al intermetallics formed in the fusion zone. The addition of the metal interlayer shows the approach for property improvement, which still obviously increases the complexity of the welding process. There, development of new adhesive composite alloy elements would be an effective way to improve the performance of the dissimilar Mg and Al joint.

## 4. Other processes

**Figure 14.**
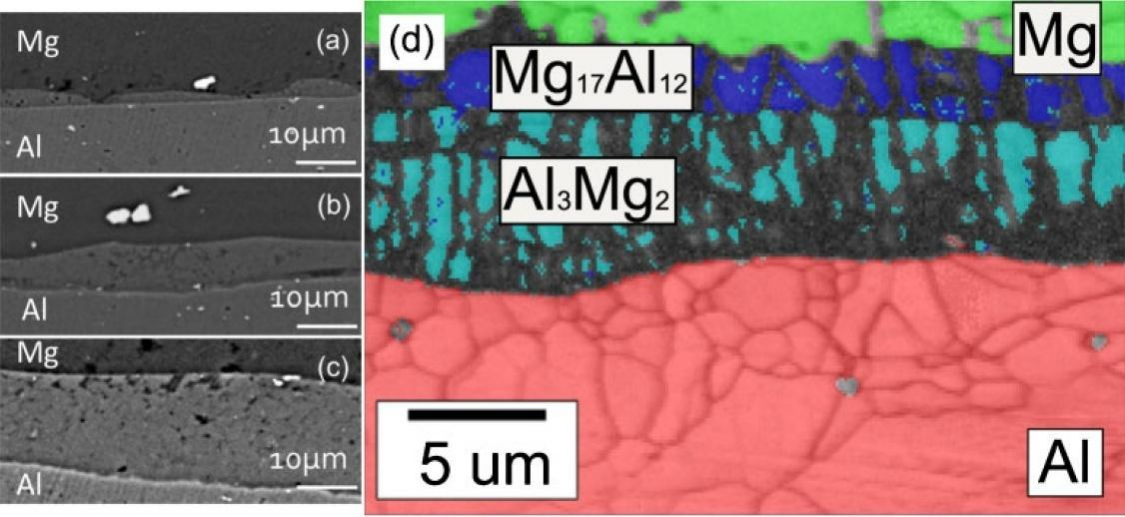
Intermetallic compound layer thickness under different parameters [84]. (**a**) 340 °C; (**b**) 370 °C; (**c**) 420 °C; (**d**) prediction.

Reaction time and energy are necessary for Mg and Al to form Mg-Al IMCs. The techniques which have a relative controllable reaction time and energy are promising for Mg and Al to obtain a high strength joint. Ultrasonic spot welding (USW) is a low heat input solid-state joining technique that may offer a solution for welding difficult dissimilar-material couples, like Mg alloys to Al alloys. Panteli *et al.* [84] found that for their optimum welding condition of 600 J (0.4 s), the reaction layer thickness was already ~5 μm thick. Intermetallic reaction centers were found to nucleate within microwelds at the interface at very short welding times and spread and grow rapidly to form a continuous layer, composed of two sub-layers of Mg_2_Al_3_ and Mg_17_Al_12_ as shown in Figure 14. Interface liquation was also found for longer welding times at temperatures below the recognized lowest eutectic reaction temperature in the AlMg binary system. Robson *et al.* [85] developed a new model to predict IMC formation during the dissimilar USW of Al and Mg alloy by ultrasonic welding. It was predicted that initial microbond formation and IMC nucleation occur early during the process, typically at 0.5 s. Once nucleated at individual microbonds, IMC islands grow by spreading and thickening under interface control, which is a rapid process and is complete within 1 s. Once neighboring islands impinge, further thickening requires diffusion through the IMC, which is a comparatively slow process.

Resistance spot welding (RSW) is the predominant welding technique in the automotive industry. The effects of parameters on the mechanical properties of resistance spot welding on Mg/Al were investigated by Hayat [86], and the result showed that the strength was negatively influenced by the formation of brittle IMCs. Ni has the high melting point (1455 °C) compared to Mg and Al, so Penner *et al.* [67] chose Ni as the interlayer, expecting that the intact remains of Ni during the RSW should entirely prevent the formation of Al-Mg intermetallics.The result showed that the formation of Al-Mg IMCs was completely suppressed using a gold coated nickel interlayer, resulting in high strength Mg/Al welds by RSW. Magnesium was joined to nickel mainly through different gold rich phases, such as residual gold coating, Mg_3_Au intermetallic compound layer and a gold-magnesium eutectic structure.

**Figure 15.**
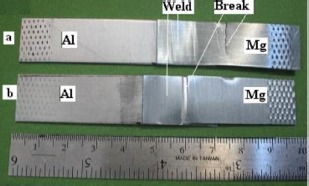
Shear tested samples of magnetic pulse welded Mg/Al joint [89].

Magnetic pulse welding (MPW) is a solid state welding technique with similarity to explosive welding [87]. The MPW process uses a magnetic field applied to an outer workpiece in order to collapse it onto the inner one at a high enough velocity to achieve a metallurgical bond. Ben-Artzy *et al.* [88] found that IMCs of different compositions were created during welding of the Al-Mg couple by rapid solidification of a thin melted layer at the interface. According to the calculated energy balance of MPW, there was enough energy to melt a thin interfacial layer and create IMC. However, the creation of IMC can be avoided using less impulse energy and/or decreasing the angle of impact of the workpieces. Kore *et al*. [89] also found there was no melting and formation of intermetallic phases at the weld interface. All their shear strength samples welded with optimum energy failed away from the weld either in the plastically deformed zone or in the base metal as shown in Figure 15.

## 5. Summary

Al and Mg alloys are potential materials used in a hybrid structure, therefore numerous studies regarding their dissimilar joining by different techniques have been undertaken. The key problem with all dissimilar metal joining techniques of Al to Mg is the easy formation of intermetallic compounds. The formation of hard and brittle IMCs has a detrimental effect on the joint strength. Using a solid state process, improving IMC variety and distribution, and reducing reaction and energy are the three main approaches to increase the Mg/Al strength. The detailed research progress and results of these techniques have been reported by many researchers.

Solid state processes which involve comparatively low welding temperatures such as friction stir welding and diffusion bonding can achieve relatively high strength. The IMC reaction layer could be significantly reduced due to the low welding temperature, but formation of brittle Al-Mg IMCs cannot be completely avoided. In order to decrease the formation of undesirable IMCs, a variety of filler metals was applied with the fusion welding processes, such as laser welding, TIG welding and laser-arc hybrid welding. Using filler metals effectively reduced the Al-Mg IMCs and improved the mechanical properties of the Al/Mg joints. Weld bonding is a combination of adhesive bonding with a welding process to gain the advantages of each joining method. In the laser weld bonding and the friction stir spot adhesive bonding process, the decomposition of the adhesive changes the flow behavior of the fusion zone and the diffusion between the Mg and Al elements, which influences the distribution and the thickness of the Mg-Al intermetallics and improves the property of the joint. Reaction time and energy are necessary for Mg and Al to form Mg-Al IMCs, so ultrasonic spot welding, resistance spot welding and magnetic pulse welding have been used because of their relatively controllable reaction time and energy. Melting and formation of Mg-Al IMCs could not be found at the weld interface in magnetic pulse welding.

## Figures and Tables

**Table 1.** Properties of pure magnesium, aluminum and iron at their melting points [13].

Properties	Magnesium	Aluminum	Iron
Ionization energy (eV)	7.6	6	7.8
Specific heat (J·kg^−1^·K^−1^)	1.36 × 10^3^	1. 08 × 10^3^	795
Specific heat of fusion (J·kg^−1^)	3.7 × 10^5^	4 × 10^5^	2.7 × 10^5^
Melting point (°C)	650	660	1.536 × 10^3^
Boiling point (°C)	1.09 × 10^3^	2.520 × 10^3^	2.860 × 10^3^
Viscosity (kg·m^−1^·s^−1^)	1.25 × 10^−3^	1.3 × 10^−3^	5.5 × 10^−3^
Surface tension (N·m^−1^)	0.559	0.914	1.872
Thermal conductivity (W·m^−1^·K^−1^)	78	94.03	38
Thermal diffusivity (m^2^·s^−1^)	3.73 × 10^−5^	3.65 × 10^−5^	6.80 × 10^−6^
Coefficient of thermal expansion (1/K)	2.5 × 10^−5^	24 × 10^−6^	1 × 10^−5^
Density (kg·m^−3^)	1.59 × 10^3^	2.385 × 10^3^	7.015 × 10^3^
Elastic modulus (N/m^3)^	4.47 × 10^10^	7.06 × 10^10^	2.1 × 10^11^
Electrical resistivity (µΩ·m)	0.274	0.2425	1.386
Vapor pressure (Pa)	360	1 × 10^−6^	2.3
